# Severely Hypoperfused Brain Tissue Correlates with Final Infarct Volume Despite Recanalization in DMVO Stroke

**DOI:** 10.5334/jbsr.3269

**Published:** 2023-11-22

**Authors:** Maud Wang, Yousra Farouki, Franny Hulscher, Benjamin Mine, Thomas Bonnet, Stephanie Elens, Juan Vazquez Suarez, Lise Jodaitis, Noemie Ligot, Gilles Naeije, Boris Lubicz, Adrien Guenego

**Affiliations:** 1 Department of Radiology, Leuven University Hospital, Leuven, Belgium; 2 Department of Interventional Neuroradiology, Erasme University Hospital, Brussels, Belgium; 3 Department of Neurology, Erasme University Hospital, Brussels, Belgium

**Keywords:** acute ischemic stroke, endovascular recanalization, distal thrombectomy, perfusion imaging

## Abstract

**Objectives:**

We sought to assess whether there were any parameter(s) on baseline computed-tomography-perfusion (CTP) strongly correlating with final-infarct-volume, and infarct volume progression after endovascular recanalization of acute ischemic stroke (AIS) with primary distal, medium vessel occlusion (DMVO).

**Materials and Methods:**

We performed a retrospective analysis of consecutive AIS patients who were successfully recanalized by thrombectomy for DMVO. By comparing baseline CTP and follow-up MRI, we evaluated the correlation between baseline infarct and hypoperfusion volumes, and final infarct volume and infarct volume progression. We also examined their effect on good clinical outcome at 3 months (defined as an mRS score of 0 to 2).

**Results:**

Between January 2018 and January 2021, 38 patients met the inclusion criteria (76% [29/38] female, median age 75 [66–86] years). Median final infarct volume and infarct volume progression were 8.4 mL [IQR: 5.2–44.4] and 7.2 mL [IQR: 4.3–29.1] respectively. TMax > 10 sec volume was strongly correlated with both (r = 0.831 and r = 0.771 respectively, p < 0.0001), as well as with good clinical outcome (–0.5, p = 0.001). A higher baseline TMax > 10 sec volume increased the probability of a higher final-infarct-volume (r^2^ = 0.690, coefficient = 0.83 [0.64–1.00], p < 0.0001), whereas it decreased the probability of good clinical outcome at 3 months (odds ratio = –0.67 [–1.17 to –0.18], p = 0.008).

**Conclusion:**

TMax > 10 sec volume on baseline CTP correlates strongly with final infarct volume as well as with clinical outcome after mechanical thrombectomy for an AIS with DMVO.

## Introduction

Endovascular thrombectomy (EVT) is now the standard of care for acute ischemic stroke (AIS) patients with an anterior large vessel occlusion (LVO) [1,2]; however, not all patients who undergo recanalization will clinically benefit from it.

Furthermore, there is still no definitive recommendation in the literature to perform EVT in patients with distal, medium vessel occlusions (DMVO) (defined by consensus as the A2–A4 segments of the anterior cerebral artery, the M3–M4 segments of the medial cerebral artery, the P2–P4 segments of the posterior cerebral artery, as well as the posterior inferior, anterior inferior, and superior cerebellar arteries [3]), despite representing up to 40% of AIS, either as primary occlusions or as secondary emboli to new territories after LVO recanalization [3,4,5,6].

Final infarct volume is a key determinant of clinical outcome [7,8,9,10], the risk of cerebral edema [11,12,13,14], and the risk of craniectomy [15,16,17], as well as that of hemorrhagic transformation [18,19,20,21] in stroke despite recanalization by EVT. Unfortunately, prediction of final infarct volume and infarct volume progression between the baseline and post-EVT imaging remains challenging in clinical practice, both in the anterior [22,23,24] and posterior [25] circulation.

Baseline imaging often includes perfusion CT as a tool to improve diagnostic accuracy [26,27], but we pondered whether the information provided by such protocols could be used as early imaging predictors of infarct volume progression. We hypothesized that the final infarct volume may be correlated with severely hypoperfused brain tissue on baseline computed tomography perfusion (CTP), regardless of successful endovascular recanalization of AIS with primary DMVO.

We sought to assess which parameter on baseline computed tomography perfusion (CTP) could be correlated with final infarct volume, infarct volume progression, as well as clinical outcome after successful endovascular recanalization of an AIS with a primary DMVO.

## Materials and Methods

This study was approved by our local institutional review board and ethics committee. Informed consent was waived based on minimal patient risk and practical inability to perform the study without the waiver.

The data that support the findings of this study are available from the corresponding author upon reasonable request. Adherence to the Strengthening the Reporting of Observational Studies in Epidemiology (STROBE) criteria was enforced [28].

### Study Population

We retrospectively identified consecutive patients diagnosed with AIS due to primary DMVO in our comprehensive stroke center between January 2018 and January 2021, who met the following criteria:

1. CTP imaging was performed in-house and provided a technically adequate assessment of the baseline infarct and hypoperfusion volumes (regional cerebral blood flow – rCBF and time-to-maximum – TMax volumes).
2. National Institutes of Health Stroke Scale (NIHSS) ≥1 at presentation.
3. Time from symptom onset to groin puncture < 6h.
4. Underwent endovascular treatment with successful recanalization, modified Treatment In Cerebral Infarction (mTICI) scale ≥2c.
5. Received follow-up magnetic resonance imaging (MRI) on day 3 post-EVT to assess the final infarct volume on fluid attenuation inversion recovery (FLAIR).
6. Clinical evaluation with modified Rankin Scale (mRS) at 3 months by a certified stroke neurologist.

The decision to perform the endovascular procedure was made by a multidisciplinary team on an individual-patient basis; the technical aspects were left at the discretion of the treating neurointerventionalist, following standard of care. Patients’ baseline clinical and radiological characteristics, procedure details, as well as outcomes, were collected using standardized definitions [29]. All cases were reviewed by two board-certified neuroradiologists with 5+ years of experience as senior staff members, they determined the angiographic treatment success using the mTICI scale. In cases of inter-observer inconsistency, a decision was made by consensus.

### Radiological Evaluation

Baseline imaging automatically assessed the infarct volume (mL; rCBF < 30% on CTP) and hypoperfusion volumes (mL; TMax > 4 sec, TMax > 6 sec, TMax > 8 sec, and TMax > 10 sec volumes) [30] before EVT using automated software (RAPID, iSchemaView, Menlo Park). CTPs with major artifacts were not included, and manual correction of the initial core lesion volumes was not required.

Final infarct volume (mL) was assessed on day 3 on axial FLAIR imaging [31] using the Horos Dicom Viewer (Horos Project, Geneva, Switzerland) by the same board-certified neuroradiologists, blinded to the initial CTP, EVT result, and clinical outcome. FLAIR axial sequences were performed on a 3T Siemens scan with the following parameters: TR 9000 msec, TE 91 msec, slice was 4mm, slice gap of 0.0, and FOV of 230 × 230mm. The readers were allowed to look at the DWI sequences to avoid misinterpretation of the FLAIR images [32].

Infarct volume progression (in mL) between baseline CTP and day 3 MRI was calculated as the difference between both infarct volumes.

### Statistical analyses

First, Pearson’s correlations were calculated to find the baseline imaging parameter (among rCBF and TMax volumes as determined on baseline CTP) with the highest correlation with final infarct volume, infarct volume progression, and 3 months good clinical outcome (mRS 0–2).

Then, we evaluated the association of this parameter with final infarct volume and infarct volume progression using linear regression, and its association with 3 months good clinical outcome using binary logistic regression.

Using receiver operating characteristic (ROC) curves analyses, we determined the optimal baseline volume threshold to predict a low final infarct volume (final infarct volume < median), a low infarct volume progression (infarct volume progression < median), and good clinical outcome (mRS 0–2 at 3 months) with optimal sensitivity and specificity.

Categorical variables are reported as proportions. Continuous variables are reported as median (inter-quartile range). Statistical significance was set at the p = 0.05 level. All statistical analyses were performed with XLSTAT (Addinsoft, New York City, NY).

## Results

**Figure 1:**
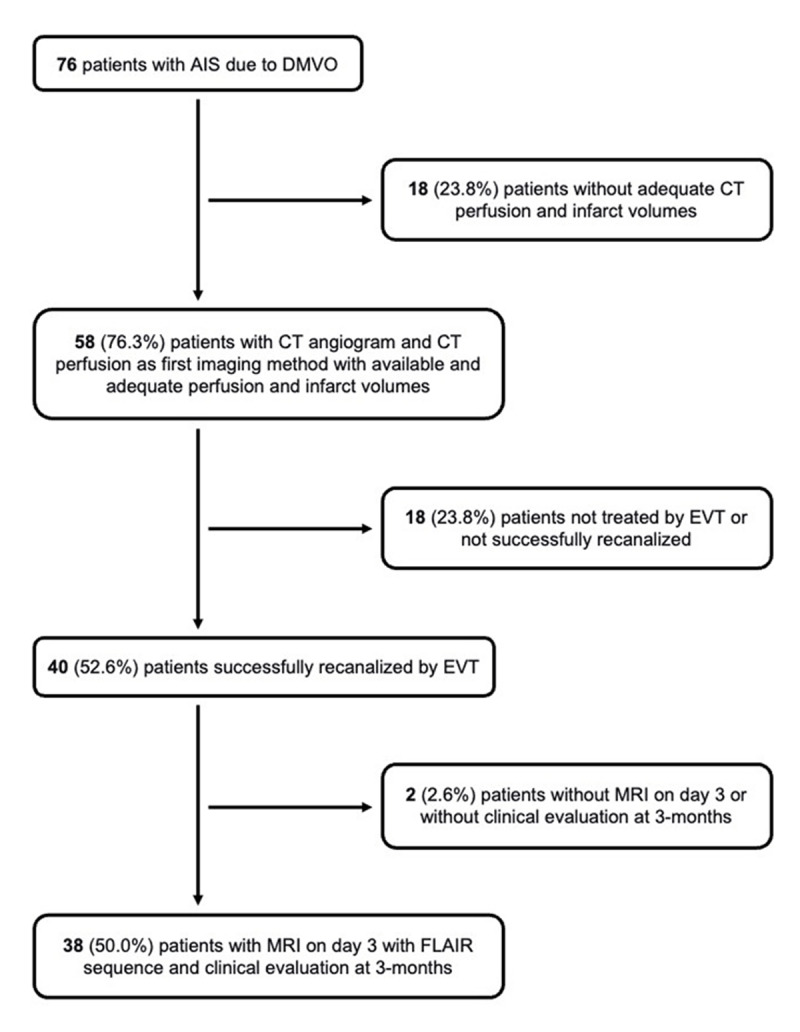
Flowchart of the patient selection.

From January 2018 to January 2021, we identified 76 consecutive patients who came into our stroke center with AIS due to DMVO. Fifty-eight patients underwent a computed tomography (CT) angiogram and CT perfusion as the first imaging method with available and adequate perfusion and infarct volumes, 40 of these patients were successfully recanalized by EVT, and 38 had magnetic resonance imaging (MRI) performed on day 3 with FLAIR sequence to assess the final infarct volume as well as a clinical evaluation at 3-months. These 38 patients were included (Figure 1).

Among them, 76% [29/38] were female, and median age was 75 [66–86] years). Baseline median infarct volume was 0.3 mL (0.3–6.8) (Table 1). Median final infarct volume and infarct volume progression were respectively, 8.4 mL [IQR: 5.2–44.4] and 7.2 mL [IQR: 4.3–29.1] (Table 2).

**Table 1:** Baseline characteristics of all patients.


	ALL

**Number of patients**	38 (100%)

**Age, years (median, IQR)**	75 (66–86)

**Female (%)**	29 (76%)

**Medical History**	

**High Blood Pressure (%)**	33 (87%)

**Diabetes (%)**	9 (24%)

**Hyperlipidemia (%)**	18 (47%)

**Weight (kg) (median, IQR)**	75 (65–94)

**Antiplatelets (%)**	12 (32%)

**Anticoagulants (%)**	7 (18%)

**Current Smoking (%)**	6 (16%)

**Pre-stroke mRS of 0–1 (%)**	36 (95%)

**Clinical Presentation**	

**Heart rate (bpm) (median, IQR)**	76 (68–88)

**Systolic Blood Pressure (mmHg) (median, IQR)**	146 (125–170)

**Diastolic Blood Pressure (mmHg) (median, IQR)**	80 (75–85)

**Temperature (°C) (median, IQR)**	36.9 (36.4–37.2)

**Baseline NIHSS (median, IQR)**	10 (8–15)

**IVT (%)**	26 (68%)

**Times**	

**Time Onset to Puncture in min (median, IQR)**	210 (166–296)

**Unknown Onset (%)**	10 (26%)

**Occlusion**	

**M2 (%)**	27 (71%)

**M3 (%)**	11 (29%)

**Left Side (%)**	20 (53%)

**Imaging**	

**Baseline infarct volume, mL (median, IQR)**	0.3 (0.3–6.8)

**TMax > 4 sec volume, mL (median, IQR)**	102 (73–126)

**TMax > 6 sec volume, mL (median, IQR)**	50 (34–76)

**TMax > 8 sec volume, mL (median, IQR)**	25 (10–48)

**TMax > 10 sec volume, mL (median, IQR)**	12.5 (0.3–37)

**Mismatch volume, mL (median, IQR)**	43.9 (30.2–58.9)

**Mismatch ratio (median, IQR)**	295 (7–488)

**Etiology**	

**Atherosclerosis (%)**	9 (24%)

**Cardio-embolic (%)**	28 (74%)

**Unknown (%)**	1 (2%)


* Continuous variables are reported as median (inter-quartile range), categorical variables as percentages.

**Table 2:** Procedural characteristics and clinical outcomes.


	ALL

**Number of patients**	38 (100%)

**Mechanical Thrombectomy**	

**Admission Mothership (%)**	20 (53%)

**Type of anesthesia**	

**General Anesthesia (GA) (%)**	37 (97%)

**Conscious Sedation then GA (%)**	1 (3%)

**Technique**	

**Contact Aspiration (%)**	13 (34%)

**Stent-retriever (%)**	6 (16%)

**Combined technique (%)**	19 (50%)

**Number of Passes (median, IQR)**	2 (1–3)

**Procedural Complication (%)**	0 (0%)

**Times**	

**Time Puncture to Recanalization in min (median, IQR)**	30 (20–55)

**Time Onset to Recanalization in min (median, IQR)**	242 (191–326)

**Early Outcomes**	

**Day 1 NIHSS (median, IQR)**	4 (1–7)

**NIHSS Shift (median, IQR)**	–3 (–9 to –1)

**Day 1 Hemorrhagic Transformation (%)**	6 (25%)

**ECASS PH-Type (%)**	2 (7%)

**Final infarct volume, mL (median, IQR)**	8.4 (5.2–44.4)

**Infarct volume progression, mL (median, IQR)**	7.2 (4.3–29.1)

**Long-Term (3 months) Outcomes**	

**mRS 0–1 (%)**	18 (47%)

**mRS 0–2 (%)**	23 (61%)

**Mortality (%)**	2 (5%)


* Continuous variables are reported as median (inter-quartile range), categorical variables as percentages.

TMax > 10 sec volume had the strongest correlation with final infarct volume and infarct volume progression (respectively, r = 0.831, p < 0.0001 and r = 0.771, p < 0.0001), as well as with good clinical outcome at 3 months (–0.5, p = 0.001).

**Figure 2:**
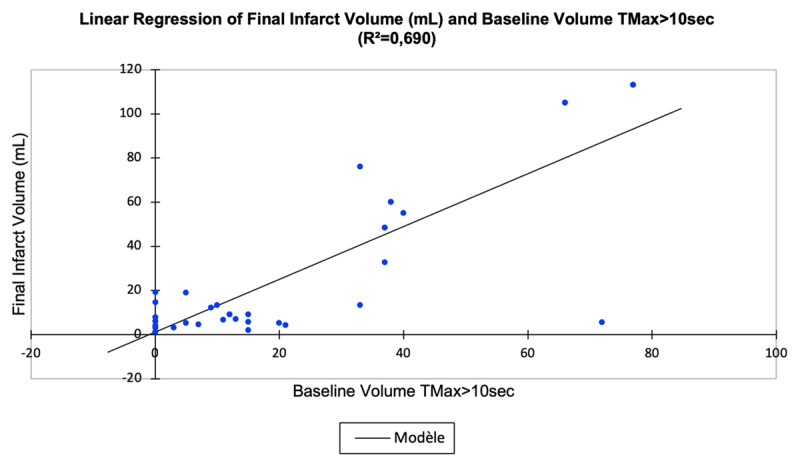
Association of baseline volume of severely hypoperfused brain tissue (TMax > 10 sec; mL) and final infarct volume (mL).

**Figure 3:**
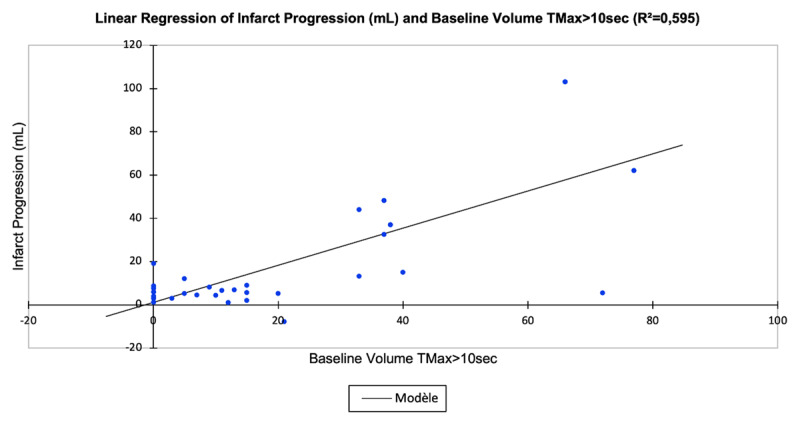
Association of baseline volume of severely hypoperfused brain tissue (TMax > 10 sec; mL) and infarct volume progression (mL).

**Figure 4:**
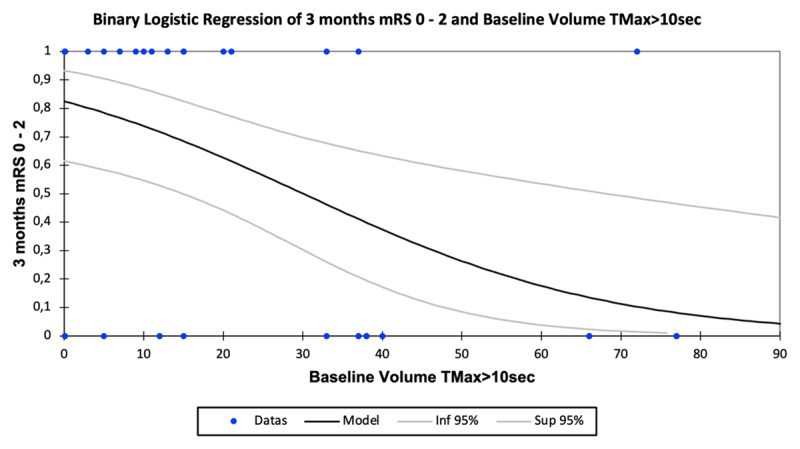
Association of baseline volume of severely hypoperfused brain tissue (TMax > 10 sec; mL) and the probability of good clinical outcome at 3 months (mRS 0-2).

A higher baseline TMax > 10 sec volume raised the probability of a higher final infarct volume (r^2^ = 0.690, coefficient = 0.83 [0.64–1.00], p < 0.0001) (Figure 2) and increased infarct volume progression (r^2^ = 0.595, coefficient = 0.77 [0.56–0.98], p < 0.0001) (Figure 3), whereas a higher baseline TMax > 10 sec volume decreased the probability of good clinical outcome at 3 months (odds ratio = –0.67 [–1.17 to –0.18], p = 0.008) (Figure 4).

ROC curves identified a TMax > 10 sec volume of less than 33 mL as the optimal threshold to predict a low final infarct volume (AUC = 0.802, sensitivity = 0.947, specificity = 0.632), a low infarct volume progression (AUC = 0.735, sensitivity = 0.947, specificity = 0.632), and good clinical outcome (AUC = 0.786, sensitivity = 0.870, specificity = 0.667).

## Discussion

Our data indicate that severely hypoperfused brain tissue defined as the TMax > 10 sec volume assessed on baseline CTP may reasonably predict final irreversibly-injured brain tissue. Patients with a low volume of severely hypoperfused brain tissue (TMax > 10 sec) had minimal infarct volume progression after successful EVT and had a higher probability of good clinical outcome.

Recently, CTP has been increasingly added to CTA in AIS evaluation for a more accurate diagnosis. Severely hypoperfused brain tissue assessed by CT perfusion has been widely used to provide a surrogate for collaterals in large vessel occlusions using the hypoperfusion intensity ratio [30,33] but has not yet extensively been evaluated in distal occlusions.

Becks at al. highlighted that CTP may improve intracranial vascular blockage detection especially in distal occlusions and posterior circulation [34]. Likewise, Amukotuwa et al. showed that TMax maps help identify infarct volume related to DMVOs [26]. Furthermore, CTP may have a prognostic role in AIS, as an association between TMax target mismatch and clinical outcomes for LVO patients has already been demonstrated [35], and CTP-derived lesion thresholds have been used to predict poor clinical outcomes or death [36].

TMax thresholds have already been evaluated for large vessel occlusions, and while Wan et al. showed an association between the hypoperfusion intensity ratios with functional outcomes [37], Seker et al. demonstrated that higher TMax volumes correlated with worse outcomes [38].

In AIS, the differentiation between core, penumbra and benign oligemia and its impact on clinical outcome still is a challenge; and severity-weighted definitions based in perfusion anomalies (as opposed to the single threshold-based concept) have already been put forward [39,40]. In patients with mild symptoms, the perfusion severity-weighted model was evaluated and showed the risk of progression of oligemic volumes to actual infarction [41,42]. Indeed, among patients with similar hypoperfusion volumes, those with proportionally larger volumes of severe TMax delays were more disposed to early neurological decline. In the Defuse 3 trial, Rao et al. demonstrated that the addition of irreversibly injured ischemic core and persistently hypoperfused tissue volumes (defined as TMax > 6sec) predicted infarct volume at day 1; other TMax thresholds however were not tested [43]. On the other hand, Fainardi et al. analyzed higher TMax thresholds in LVO patients selected for mechanical thrombectomy and demonstrated that all TMax parameters were predictors of final infarct volume and outcome but that the higher thresholds ( > 6 seconds) had the strongest association with final infarct volume and good outcome [36]. A recently published study evaluated multiple TMax delays in DMVO patients with failed recanalization, and found that longer TMax delays, with the preeminence of TMax > 10 sec, were best correlated to final infarct volume [44].

Our preliminary data have the potential to guide further research in identifying non-salvageable tissue before EVT, and in determining imaging protocols for future trials involving DMVO stroke patients. Identifying salvageable vs. irreversibly damaged brain tissue, and thus determining which patients would benefit most from EVT remains an important consideration in the treatment of acute stroke especially in difficult situations such as DMVOs. This could have implications in the management of those patients, such as the need of repeating brain imaging in case of delayed transfer, as well as prognosis evaluation and clinical and imaging follow-up.

Our findings are the results of a retrospective, observational study with inherent biases such as single-center design and small sample size. Therefore, these findings are preliminary, hypothesis-generating, and do not allow for firm conclusions. Consequently, these data need to be replicated in larger cohorts as well as for patients with unsuccessful recanalization.

Concerning the evaluation of recanalization results, one would prefer to solely use the mTICI 3 score instead of mTICI 2c, however it would not be technically feasible in DMVO to go for a mTICI 3 after reaching a mTICI 2c considering the distality of the remaining occlusions.

The final infarct volume was estimated from MRI scans obtained three days after symptom onset. Ideally, infarct growth should be determined by the same imaging modality detecting irreversibly injured brain tissue at baseline and during follow-up, unfortunately no imaging modality would display the exact ischemic brain infarction within the first 6 hours of stroke onset. Non-contrast CT hypoattenuation represents ionic edema, diffusion impairment on DWI depicts extracellular space shrinkage and consecutive cellular edema, showing brain areas with neuronal function failure, but does not exactly differentiate reversible and irreversible injury [45].

We did not calculate an infarct-growth rate (volume difference between follow-up infarct volume and baseline infarct volume divided by the time period between both images) because of our small sample size and relatively large proportion of patients without clear onset.

Lastly, final infarct volume assessment on FLAIR at day 3 post-stroke onset may be restricting [32] as ischemia is a dynamic process of cytotoxic, ionic, and vasogenic edema, and the influence of other factors on brain hypoperfusion has yet to be addressed in larger prospective studies. Yet, all patients underwent the same process and readers were allowed to evaluate the DWI sequence at the same time.

## Conclusions

This preliminary study suggests that severely hypoperfused brain tissue defined as TMax > 10 sec volume on baseline CTP is strongly associated with final infarct volume and progression as well as clinical outcome after EVT recanalization of AIS due to DMVO.
